# RNA mis‐splicing in children with congenital myotonic dystrophy is associated with physical function

**DOI:** 10.1002/acn3.52224

**Published:** 2024-10-25

**Authors:** Julia M. Hartman, Kobe Ikegami, Marina Provenzano, Kameron Bates, Amanda Butler, Aileen S. Jones, Kiera N. Berggren, Jeanne Dekdebrun, Marnee J. McKay, Jennifer N. Baldwin, Kayla M. D. Cornett, Joshua Burns, Michael Kiefer, Nicholas E. Johnson, Melissa A. Hale, the DMCRN Consortium

**Affiliations:** ^1^ Medical Scientist Training Program Virginia Commonwealth University Richmond Virginia 23298 USA; ^2^ Center for Inherited Myology Research Virginia Commonwealth University Richmond Virginia 23298 USA; ^3^ Department of Neurology Virginia Commonwealth University Richmond Virginia 23298 USA; ^4^ Department for Human and Molecular Genetics Virginia Commonwealth University Richmond Virginia 23298 USA; ^5^ Children's Hospital of Richmond at Virginia Commonwealth University Pediatric Therapy Services Richmond Virginia 23220 USA; ^6^ Department of Neurology University of Rochester School of Medicine and Dentistry Rochester New York 14642 USA; ^7^ Sydney School of Health Sciences, Faculty of Medicine and Health The University of Sydney Sydney New South Wales 2006 Australia; ^8^ Sydney Children's Hospitals Network (Randwick and Westmead) Sydney New South Wales Australia; ^9^ Department of Physical Therapy Virginia Commonwealth University Richmond Virginia 23298 USA

## Abstract

**Objectives:**

Dysregulated RNA alternative splicing is the hallmark of myotonic dystrophy type 1 (DM1). However, the association between RNA mis‐splicing and physical function in children with the most severe form of disease, congenital myotonic dystrophy (CDM), is unknown.

**Methods:**

Eighty‐two participants (42 adults with DM1 and 40 children with CDM) with muscle biopsies and measures of myotonia, motor function, and strength were combined from five observational studies. Data were normalized and correlated with an aggregate measure of alternative splicing dysregulation, [MBNL]_inferred_, in skeletal muscle biopsies. Multiple linear regression analysis was performed to predict [MBNL]_inferred_ using clinical outcome measures alone. Similar analyses were performed to predict 12‐month physical function using baseline metrics.

**Results:**

Myotonia (measured via vHOT) was significantly correlated with RNA mis‐splicing in our cross‐sectional population of all DM1 individuals; CDM participants alone displayed no myotonia despite a similar range of RNA mis‐splicing. Measures of motor performance and muscle strength were significantly associated with [MBNL]_inferred_ in our cohort of all DM1 individuals and when assessing children with CDM independently. Multiple linear regression analyses yielded two models capable of predicting [MBNL]_inferred_ from select clinical outcome assessments alone in all subjects (adjusted *R* ^2^ = 0.6723) or exclusively in children with CDM (adjusted *R* ^2^ = 0.5875).

**Interpretation:**

Our findings establish significant correlations between skeletal muscle performance and a composite measure of alternative splicing dysregulation, [MBNL]_inferred_, in DM1. The strength of these correlations and the development of predictive models will assist in designing efficacious clinical trials for individuals with DM1, particularly CDM.

## Introduction

Myotonic dystrophy type 1 (DM1) is the most common form of muscular dystrophy with a prevalence of 1 in 2100.^1^ DM1 is inherited in an autosomal dominant manner and is caused by a CTG trinucleotide repeat expansion (CTG_n_) in the 3′ untranslated region of the dystrophia myotonica protein kinase (*DMPK*) gene.^2^, ^3^, ^4^ Although nearly every organ system can be affected, the core clinical features of DM1 include progressive distal muscle weakness, early onset cataracts, and myotonia (i.e., delayed muscle relaxation following contraction).^5^, ^6^

Congenital myotonic dystrophy (CDM) is the most severe form of DM1 and results from large, intergenerational CTG_n_ expansion between parent and child.^7^, ^8^, ^9^ CDM presents at birth with symptoms of hypotonia, clubfoot, feeding difficulties, and respiratory distress.^10^, ^11^, ^12^ In contrast to adults with DM1 who present with a consistent, progressive decline in muscle function over time, children with CDM exhibit a natural improvement and stabilization of motor function and muscle performance in early childhood. As children with CDM progress through adolescence and into early adulthood, symptoms become more consistent with adult‐onset DM1.^12^, ^13^

In DM1, the core pathogenesis is the sequestration of muscleblind‐like (MBNL) RNA binding proteins by toxic, expanded CUG_n_ RNAs within nuclear aggregates termed foci. This leads to an overall reduction in the functional concentration of MBNL in affected tissues and subsequent dysregulation of RNA metabolism.^14^, ^15^ As critical regulators of fetal to adult mRNA isoform transitions, depletion of functional MBNL levels leads to global perturbations in splicing regulation and reversion of transcripts to the fetal isoform in affected tissues.^16^, ^17^, ^18^, ^19^, ^20^ Numerous transcripts are mis‐spliced, although only a select few have been linked to disease phenotypes in DM1 cell and animal models, including *CLCN1* and myotonia,^21^, ^22^ *SCN5A* and cardiac arrhythmia,^23^ and *BIN1* and muscle weakness.^24^

While RNA mis‐splicing of individual events has been correlated with ankle dorsiflexion performance and manual muscle testing in individuals with adult‐onset DM1,^25^, ^26^ this relationship has not been replicated using other measures of physical performance. Additionally, no such associations have been evaluated in children with CDM. Previous work by our group has characterized global splicing dysregulation in skeletalmuscle of DM1 adults and a cohort of children with CDM using a composite measure of MBNL‐dependent splicing, [MBNL]_inferred_. This representative metric of free intracellular MBNL concentration strongly correlates with global splicing dysregulation as captured by total RNA sequencing.^27^, ^28^

Using this data, we aimed to investigate if transcriptome‐wide RNA mis‐splicing as measured by [MBNL]_inferred_ correlates with a wider breadth of functional measures assessing myotonia, motor function, and strength in a cross‐sectional cohort of adults with DM1 and children with CDM. Additionally, we sought to evaluate if [MBNL]_inferred_ levels in individuals with CDM correlate with the patterns of functional improvement observed throughout childhood in this affected population. Using these correlative analyses, we developed regression models to predict [MBNL]_inferred_ using measures of physical function. This model may offer a noninvasive alternative for predicting disease‐associated mis‐splicing in affected individuals. Lastly, we developed regression models to predict 12‐month physical function using baseline performance and baseline [MBNL]_inferred_ values.

## Methods

### Study design

Clinical phenotypes and associated biopsies from five multisite longitudinal observational studies were used to determine the correlation between splicing dysregulation (as measured by [MBNL]_inferred_) and physical function in a cohort of children with CDM and adults with DM1. Muscle biopsies were collected at a baseline visit. Clinical assessments were completed at baseline and at a 3‐month visit and/or at a 12‐month visit depending on the study they were enrolled in. Participants provided informed consent before enrollment per the local site's Institutional Review Board. For participants under 18, written informed consent was obtained from one parent and verbal assent from children over the age of 8.

Children with CDM between the ages of 0–17 (inclusive) were enrolled in the HELP‐CDM, TREAT‐CDM, or ASPIRE studies (NCT03059264, NCT05224778).^13^ Diagnostic criteria for CDM were defined as symptoms of myotonic dystrophy in the newborn period (<30 days) including hypotonia, respiratory distress, feeding difficulty, or clubfoot requiring hospitalization greater than 72 h, and a genetic test confirming an expanded trinucleotide CTG_n_ repeat in the *DMPK* gene (CTG repeats greater than 200) or an affected mother. Exclusion criteria were described previously.^13^

Adults with DM1 over the age of 18 were enrolled in HELP‐DM1 or END‐DM1 studies (NCT03981575). Participants were enrolled if they had a clinical diagnosis of DM1 or a positive genetic test confirming an expanded CTG repeat in the *DMPK* gene. They were excluded if they had symptomatic renal or liver disease, uncontrolled diabetes mellitus or thyroid disorders, or were pregnant. Mexiletine or other anti‐myotonia agents were required to be stopped at least 72 hours prior to a study visit.

**Figure 1:**
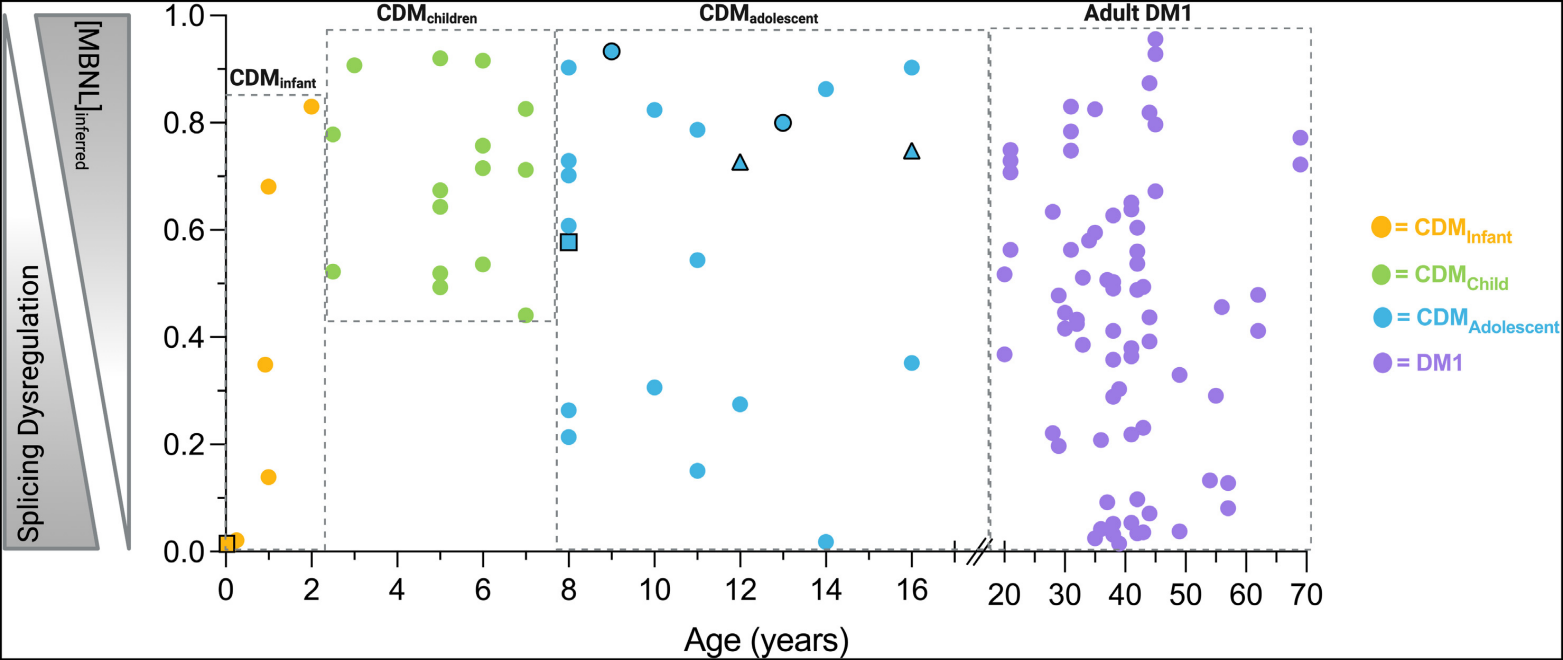
[MBNL]_inferred_ values calculated from DM1 and CDM skeletal muscle total RNA sequencing across pediatric development and adulthood. [MBNL]_inferred_ values range from 0 to 1 and inversely correlate with global mis‐splicing severity. CDM infants (CDM_infant_; ≤ 2 years) are shown in yellow. CDM children (CDM_child_; 2–8 years) are shown in green; CDM adolescent (CDM_adolescent_; 8–16 years) individuals are show in blue. Adult DM1 (20–69 years) individuals are shown in purple. CDM‐01 longitudinal biopsies shown as black‐outlined squares. CDM‐30 longitudinal biopsies shown as black‐outlined circles. CDM‐37 longitudinal biopsies are shown as black‐outlined triangles.

Select participants provided a muscle biopsy as part of an in‐house biorepository study. While clinical assessments were not available for these participants, [MBNL]_inferred_ was calculated and these values were used to further illustrate the dynamic range of mis‐splicing observed across the lifespan in Figure 1.

### Clinical assessments

Myotonia was assessed viaparticipant (or caregiver if under 12) report and clinical observation. Participants rated their myotonia severity on a 6‐point scale from “1, I don't experience this” to “6, It affects my life severely” as part of the Myotonic Dystrophy Health Index (MDHI) or Congenital and Childhood Myotonic Dystrophy Health Index (CCMDHI).^29^ Clinical myotonia was observed using video hand opening time (vHOT),^30^, ^31^ in which the time to extend the thumb after 4 sec of maximal hand flexion contraction is calculated via video recording of the procedure. The assessment was modified for children to squeeze a rubber toy for improved understanding of the task and time was scored live using a stopwatch.

Motor function measures included the 9‐hole peg test,^32^ 6‐min walk,^33^, ^34^ 4 stair climb,^35^ and 10‐meter walk/run test^35^ administered according to previously described methods. For adults enrolled in HELP‐DM1, a 10‐stair climb with railing as described in the modified dynamic gait index (mDGI) was performed.^36^

Strength measures for knee extension,^37^ grip,^13^ and ankle dorsiflexion^13^ were assessed via Quantitative Myometry (QMT) according to previously published methods. In adults, a fixed QMT system with a force transducer attached to a metal frame was used to provide adequate stabilization due to high force outputs. In children, handheld dynamometry was used to reduce the administration burden. Strength was measured in kilogram‐force (kgf) units and converted to Newtons (N) by standard conversion of 9.80665 (e.g., 1 kgf = 9.80665 N).

### Muscle biopsy collection and derivation of [MBNL]_inferred_

Muscle biopsies from adult DM1 participants were collected with either a Bergstrom or 14‐gauge argon Supercore needle of the tibialis anterior (TA) as previously described.^38^ Muscle biopsies of the vastus lateralis from CDM children were obtained using either an open surgical technique or a 14‐gauge Argon Supercore needle.^28^ In all studies, biopsies were not performed in individuals with known bleeding disorders, a history of anti‐coagulation medication use, or a platelet count less than 50,000. To participate in the muscle biopsy, ankle dorsiflexion strength had to be between 4+ and 4− on the Medical Research Council (MRC) scale for muscle strength.

Total RNA sequencing was performed on RNA extracted from all muscle biopsies, and [MBNL]_inferred_ values were derived for each individual as previously described.^27^, ^28^ In brief, percent‐spliced in (PSI) values from 9 skipped exon splicing events with high predictive power of overall global mis‐splicing in DM1 muscle were used to calculate [MBNL]_inferred_.^27^, ^28^ Given that we were utilizing additional samples, [MBNL]_inferred_ values were recalculated using all participants. CDM participant sub‐cohort classifications were previously defined and based on age at biopsy.^28^ The CDM sub‐cohorts are defined as (i) CDM_infant_ (≤2 years), (ii) CDM_child_ (>2–8 years), and (iii) CDM_adolescent_ (≥8 years).^28^ Individual PSI values for *CLCN1* exon 7a and *CACNA1S* exon 29 events were derived from comparisons between each affected group (CDM sub‐cohort or DM1 adults) and associated age‐matched, unaffected individuals as previously reported.^28^

### Statistical analyses

Motor function and strength measures were converted to percent predicted of sex and age‐matched healthy controls for analysis. Percent predicted for walk/running speed was calculated using previously published data^39^, ^40^ and percent predicted for the 6‐min walk test, stair climb speed, 9‐hole peg test, grip strength, ankle dorsiflexion, and knee extension were calculated using raw data from the 1000 Norms Project.^41^, ^42^

Study data were collected and managed using REDCap® electronic data capture tools^43^, ^44^ and exported to Microsoft Excel Version 16.75 for analysis. Statistical analyses were performed using GraphPad Prism 10.1.0, and *p*‐values <0.05 were considered significant. Clinical data points inconsistent with the cohort distribution were verified with the original data sources by the clinical evaluators and subsequently reviewed by the study's principal investigator (P.I.).

Univariate correlations and multiple linear regression were performed in GraphPad Prism. Univariate Spearman correlations assumed data are not sampled from Gaussian distributions with a two‐tailed p‐value and a 95% confidence interval. Given that myotonia was measured using a self−/parent‐reporting scale, data were assumed to be nonparametric. Responses were accumulated between the various DM1 subgroups, and statistical significance between the group medians was evaluated using a Kruskal–Wallis test. Dunn's test was used to correct for multiple comparisons. To test for the overall difference between total adult DM1 and CDM myotonia scale responses, a Mann–Whitney test was used that assumed non‐Gaussian distributed data with a two‐tailed *p*‐value.

Multiple linear regression analysis assumed a least squares regression, and adjusted R‐squared was used to quantify goodness‐of‐fit. D'Agostino‐Pearson omnibus normality test, Anderson‐Darling test, Shapiro–Wilk normality test, and the Kolmogorov–Smirnov normality test with Dallal–Wilkinson–Lillie for p‐value were all performed alongside multiple linear regression analysis to ensure data normality. A 95% confidence level was used.

## Results

### Evaluation of [MBNL]_inferred_ in skeletal muscle biopsies from children with CDM and adults with DM1

We had previously used total RNA sequencing to characterize MBNL‐dependent splicing dysregulation in skeletal muscle samples from subjects with CDM and DM1 and calculated [MBNL]_inferred_, an aggregate metric of RNA mis‐splicing representative of estimated intracellular concentrations of free MBNL. [MBNL]_inferred_ values range from 0 to 1, with 0 indicating high MBNL depletion consistent with significant mis‐splicing and 1 representative of intracellular MBNL concentrations comparable to unaffected individuals. Using this methodology, we identified a triphasic modality of RNA mis‐splicing progression across pediatric development within our cross‐sectional cohort of children with CDM which appeared to mirror the triphasic phenotypic progression observed.^28^ In brief, individuals with CDM under the age of 2 (CDM_infant_) presented with severe mis‐splicing consistent with the severity of disease presentation at birth. This is followed by a universal and significant reversal of splicing dysregulation in early childhood (CDM_child_, 2–8 years). This observation is supported by the longitudinal sampling of CDM‐01 at 2 weeks and 8 years of age where a marked increase in [MBNL]_inferred_ was observed ([MBNL]_inferred_ = 0.015 and 0.577, respectively). RNA mis‐splicing universally improved in early childhood (CDM_child_, 2–8 years) with some individuals approaching splicing patterns like that of unaffected age‐matched individuals. Sampled adolescent children displayed a spectrum of [MBNL]_inferred_ values, indicating a wide range of global splicing dysregulation. Post 8 years of age, a gradient of [MBNL]_inferred_ in CDM_adolescent_ was observed and mirrors that of individuals with adult‐onset DM1, for which a full range of splicing dysregulation is observed. This triphasic pattern of RNA mis‐splicing throughout pediatric development in children with CDM is visualized in Figure 1.

To assess potential correlations between skeletal muscle performance measures and alternative splicing dysregulation, we aggregated CDM and DM1 subjects that (i) received a muscle biopsy, (ii) had a [MBNL]_inferred_ score derived from matched skeletal muscle RNA, and (iii) possessed clinical outcomes from the same associated visit. While a total of 116 muscle biopsies were available from 82 unique participants across the five natural history studies and an in‐house biorepository study (Fig. 1, Table S1), only 101 of the biopsies had associated clinical outcome data (n = 31 CDM & 70 DM1, Table S2). The finalized cross‐sectional cohort that we used for the analyses herein contains participants with time point matched [MBNL]_inferred_ scores and clinical outcomes from either baseline visits (children and adults), 3‐month visits (adults only), or sporadic longitudinal sampling (three children with CDM only, indicated in Fig. 1 and Table S1). Additional clinical information from participants at 12‐month visits was collected for predictive modeling even though there was no associated muscle biopsy, and therefore no available [MBNL]_inferred_ score, from that timepoint (Tables S1 and S2). One sample (CDM‐38) was excluded from data analysis as the biopsy was collected from the soleus. Baseline visit characteristics including mean [MBNL]_inferred_ and measures of myotonia, motor function, and strength are presented in Table 1 for all disease subcohorts. Raw clinical outcome data for all participants at all biopsy‐matched timepoint sampling can be found in Table S2.

**Table 1:** Characteristics of CDM and DM1 participants at baseline visit.

Baseline characteristics	CDM			DM1
	Infant	Child	Adolescent	Adults
Sample size (*n*)	7	15	20	42
Biological sex (*n*)				
Male	6	11	9	17
Female	1	4	11	25
Age (years)	0.76 ± 0.7	5.2 ± 1.5	10.9 ± 2.9	39.3 ± 10.2
CTG repeat length	1600 ± 141.4	1035 ± 334.6	1191 ± 540.6	426 ± 253.5
[MBNL]_inferred_	0.29 ± 0.3	0.69 ± 0.2	0.61 ± 0.3	0.47 ± 0.2
Biopsy site	Vastus lateralis	Vastus lateralis	Vastus lateralis	Tibialis anterior
Clinical outcome measures				
Myotonia Scale	1	2.1 ± 1.4	2.4 ± 1.5	3.2 ± 1.5
Myotonia vHOT_thumb_ (s)	NA	0.5 ± 0.2	1.1 ± 1.1	7.5 ± 8.4
6‐minute walk (m)	NA	338 ± 91	365± 140	377 ± 103
Walk/running speed (m/s)	NA	1.9 ± 0.6	2.1 ± 0.74	1.99 ± 0.9
Stair climb speed (stairs/s)	NA	1.2 ± 0.4	1.2 ± 0.7	1.7 ± 0.7
9‐hole peg test (s)	NA	47.7 ± 14.7	44.6± 31.9	20.1 ± 6.6
Grip strength (kgf)	NA	4.1 ± 1.5	7.1 ± 5	13.9 ± 8.5
Ankle dorsiflexion (kgf)	NA	5.0 ± 2.9	6 ± 3.7	8 ± 5.4
Knee extension (kgf)	NA	8.2 ± 2.6	9.6 ± 3.8	22.1 ± 9.0

Mean ± one SD reported unless otherwise indicated.

kgf, kilogram‐force; m, meters; *n*, number of samples; s, seconds.

### RNA mis‐splicing correlates with myotonia measures in all participants with DM1 but not in children with CDM alone

**Figure 2:**
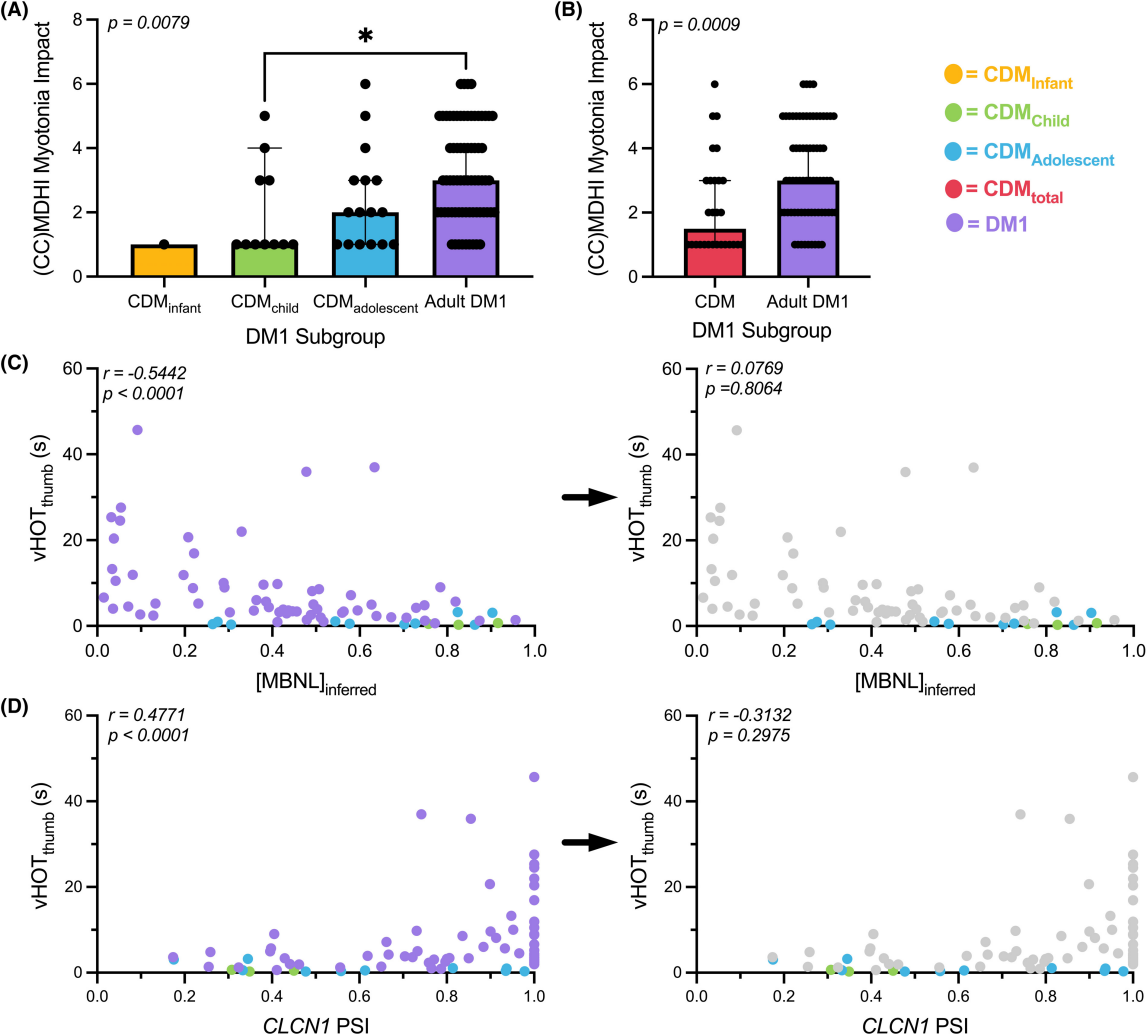
Myotonia measures in adult DM1 and CDM participants correlate disparately with skeletal muscle spliceopathy. (A) Myotonia MDHI/CCMDHI scale average responses across CDM subcohorts and adult DM1 participants. Results are expressed as median ± 95% confidence interval (CI) via Kruskal–Wallis test with Dunn's multiple comparisons test. (B) Myotonia scale average value between DM1 individuals and all CDM individuals. Results are expressed as median ± 95% CI via unpaired Mann–Whitney test. (C) Correlation between [MBNL]_inferred_ and vHOT_thumb_ times in all DM1 individuals (left) and CDM individuals alone (right panel). Adult DM1 measures are removed from the statistical analysis in the right panel but grayed out and included in the figure for visual reference. (D) Correlation between *CLCN1* exon 7a percent spliced in (PSI) and vHOT_thumb_ times in all DM1 individuals and CDM individuals. All correlations are reported from a two‐tailed Spearman test.

Myotonia was assessed via two methods – a qualitative participant/caregiver reported score of impact on daily living (from MDHI/CCHDMI) and quantitatively via video of hand opening time (vHOT). The impact of myotonia on a participant's life via participant/caregiver reported scale was found to be significantly different between all groups (p = 0.0079) (Fig. 2A). Given the large observed differences in mean [MBNL]_inferred_ values between CDM subcohorts (Table 1), it is reasonable to infer that [MBNL]_inferred_ values correlate minimally with the impact of myotonia on a participant's life (Fig. 2A). Multiple comparisons testing revealed a significant difference between CDM_child_ responses (median response ≅ 1) and adult DM1 responses (median response ≅ 3) to the myotonia scale (*p* = 0.0498) (Fig. 2A). When comparing the values of the self‐reported scale in adult DM1 individuals to total CDM responses (CDM_total_), a significant increase in perceived impact of myotonia was observed for adults with DM1 (Fig. 2B).

vHOT was used to quantitatively evaluate clinical myotonia between the DM1 subgroups. vHOT_thumb_ was found to correlate moderately with [MBNL]_inferred_ in all individuals with DM1 (Spearman *r* = −0.54) (Fig. 2C, left). However, there was no significant correlation observed when examining just participants with CDM despite the comparable range of spliceopathy observed (Spearman *r* = 0.08) (Fig. 2C, right). To further investigate this phenomenon, we directly examined the relationship between vHOT and *CLCN1* exon 7a inclusion (percent spliced in, PSI). Defects in skeletal muscle chloride conductance due to mis‐splicing of *CLCN1* are postulated to be causative of myotonia and restoration of aberrant exon 7a inclusion corrects this phenotype in DM1 mouse models.^45^ Consistent with this pathogenic mechanism, *CLCN1* PSI was significantly correlated with vHOT_thumb_ times in all individuals with DM1 (Spearman *r* = −0.48). In contrast, this relationship was not observed in participants with CDM alone (Fig. 2D, right). While select CDM_adolescent_ individuals have nearly 100% *CLCN1* exon 7a inclusion, like that of their adult DM1 counterparts with the highest vHOT_thumb_ times, minimal myotonia was observed. Mis‐splicing of *CACNA1S* (CaV1.1), a calcium channel that controls skeletal muscle excitation–contraction coupling, has also been associated with exacerbated myopathy and myotonia in DM1.^46^, ^47^ In our complete cross‐sectional cohort, reduced *CACNA1S* exon 29 inclusion was significantly negatively correlated with longer vHOT_thumb_ times, but not in participants with CDM alone (Fig. S1). Overall, the lack of correlation between CDM vHOT_thumb_ times and either a composite measure of disease‐associated spliceopathy or mis‐splicing of specific, phenotype‐associated events is driven by the lack of quantifiable myotonia within children with CDM throughout development. This is consistent with previous observations that individuals with CDM within the first decade of life do not experience clinical myotonia.^48^ However, our analysis is the first of its kind to demonstrate that this phenomenon occurs even when phenotype‐associated RNA mis‐splicing occurs at levels comparable to adults with DM1 who present with severe myotonia.

### RNA mis‐splicing correlates moderately with select motor function measures in all participants with DM1

We next assessed whether global mis‐splicing as measured by [MBNL]_inferred_ correlated with several timed motor tests within all DM1 participants or selectively within children with CDM, as the utility of certain clinical outcome measures may vary with age and disease severity.^49^

**Figure 3:**
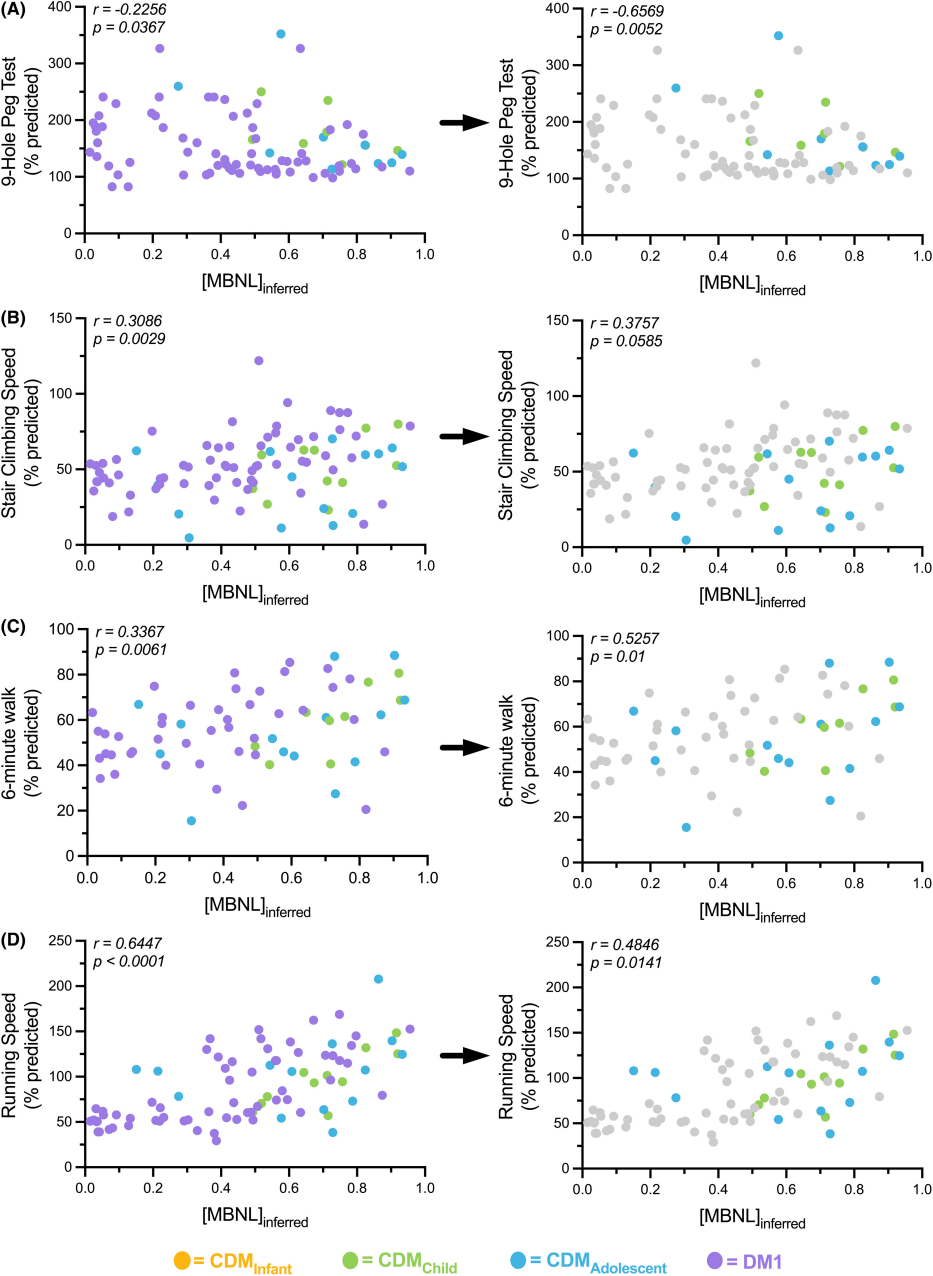
MBNL‐dependent mis‐splicing as measured by [MBNL]_inferred_ correlates moderately with motor function measures in DM1 individuals. Correlation between [MBNL]_inferred_ and (A) 9‐hole peg test (% predicted), (B) stair climbing speed (% predicted), (C) 6‐min walk distance (% predicted), and (D) walk/running speed (% predicted) in all DM1 individuals and CDM individuals. Left panels represent correlations of all DM1 subjects and right panels represent CDM subjects only. The adult DM1 measures were excluded from the CDM statistical analyses but grayed out and included in the figure on the right for visual reference. Correlations are reported from a two‐tailed Spearman test.

The 9‐hole peg test was significantly correlated, albeit weakly, with [MBNL]_inferred_ values in all individuals with DM1 (Spearman *r* = −0.23). The relative association significantly improved in children with CDM alone whereby individuals with higher [MBNL]_inferred_ values were able to perform the test much quicker with results more in line with control times (i.e., resulting in a lower % predicted) (Spearman *r* = −0.66) (Fig. 3A). In contrast to the 9‐hole peg test, weak to moderate positive correlations were observed for both 6‐minute walk distance and stair climbing speed in all participants with DM1 and in children with CDM alone (Fig. 3B,C). The strongest correlation observed between any motor function measure and [MBNL]_inferred_ was 10‐meter walk/running speed. Percent predicted walk/running speed had a strong positive association with [MBNL]_inferred_ levels in all individuals with DM1 that was only minimally reduced when assessing participants with CDM independently (Fig. 3D).

### [MBNL]_inferred_ correlates strongly with skeletal muscle strength measures in all participants with DM1

**Figure 4:**
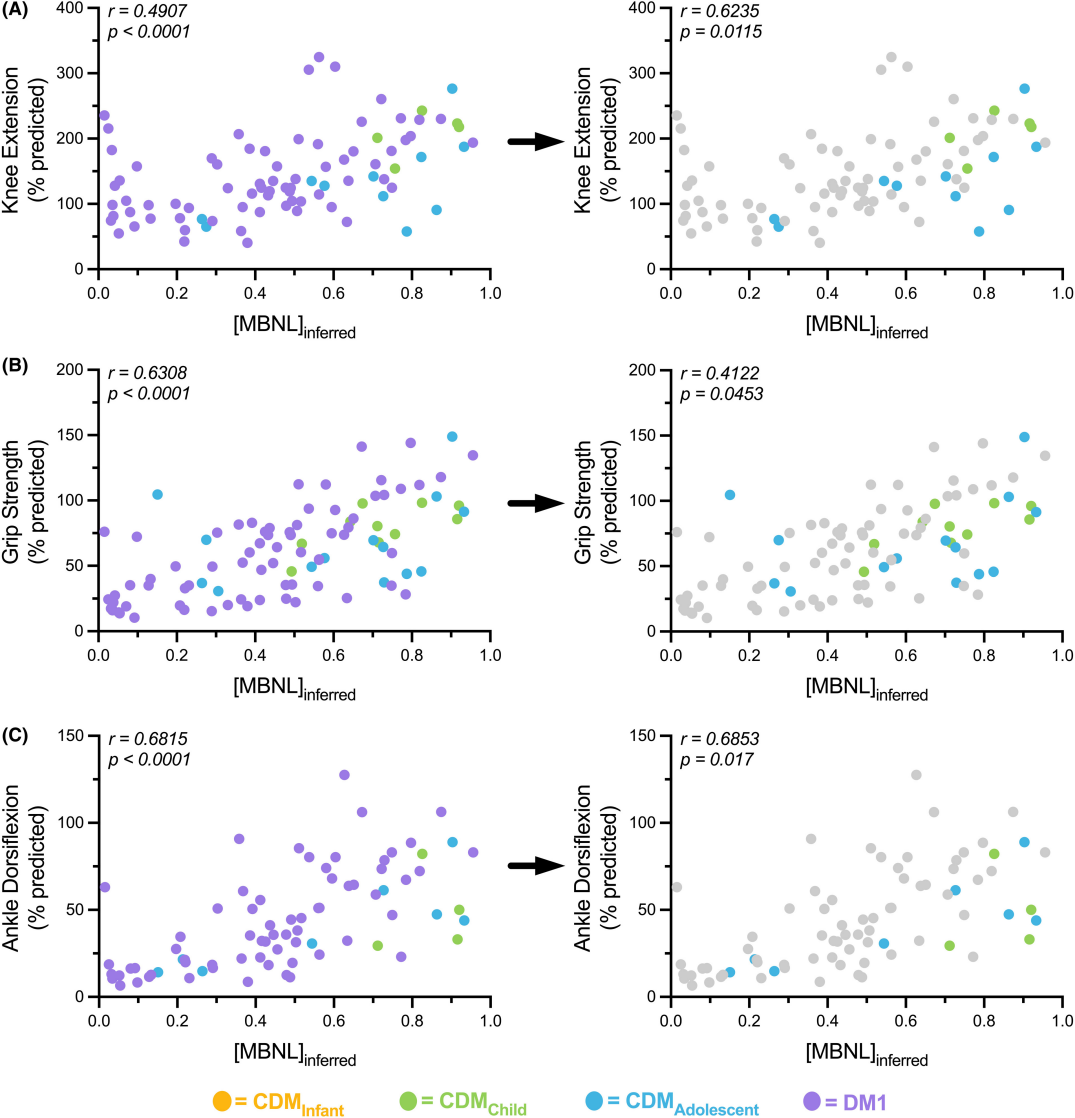
MBNL‐dependent mis‐splicing as measured by [MBNL]_inferred_ correlates with muscle strength independent of age. Correlation between [MBNL]_inferred_ and (A) knee extension (% predicted), (B) hand grip strength (% predicted), and (C) ankle dorsiflexion (% predicted) in all DM1 and CDM individuals. Left panels represent correlations of all DM1 subjects and right panels represent CDM subjects only. The adult DM1 measures were excluded from the CDM statistical analyses but grayed out and included in the figure on the right for visual reference. Correlations are reported from a two‐tailed Spearman test.

Quantitative muscle testing of knee extension (KE), hand grip (HG), and ankle dorsiflexion (ADF) strength had the strongest associations with [MBNL]_inferred_ levels compared to all other outcome assessments utilized in these analyses. When adults with DM1 and children with CDM were evaluated in combination, higher levels of [MBNL]_inferred_ were strongly correlated with muscle strength; the highest reported association was with ADF (Spearman *r* = 0.68) (Fig. 4). The strength of these relationships was generally maintained in CDM individuals alone (Fig. 4). Strikingly, the relative association of [MBNL]_inferred_ with ADF in children with CDM was identical to that observed in all participants with DM1 (Spearman *r* = 0.69), in part due to the range of ADF performance captured in CDM_adolescent_ participants that replicated the range observed in adults (Fig. 4C, blue and purple samples, respectively). Consistent with previous reports of correlations between RNA mis‐splicing and muscle strength, we found that measures of strength correlate strongly with [MBNL]_inferred_ in this assembled DM1 cohort.

### [MBNL]_inferred_ can be predicted using physical function measures alone

**Figure 5:**
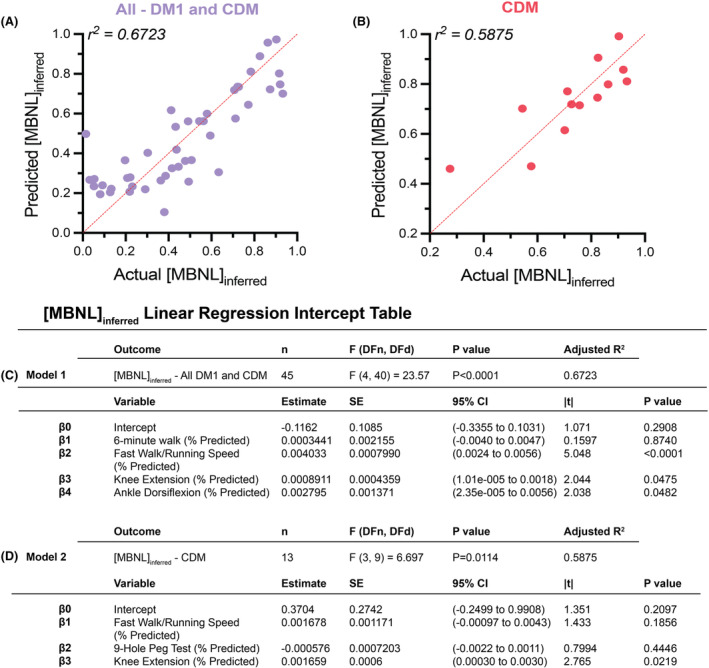
Multiple linear regression modeling to predict [MBNL]_inferred_ in DM1 participants using functional outcomes. (A) Multiple linear regression plot showing observed [MBNL]_inferred_ versus predicted [MBNL]_inferred_ for all DM1 individuals. (B) Multiple linear regression plot showing observed [MBNL]_inferred_ versus predicted [MBNL]_inferred_ for CDM individuals. Intercept table of multiple linear regression models for (C) complete DM1 cohort and (D) CDM cohort alone using clinical outcome measures to predict [MBNL]_inferred_. Multiple statistical elements are reported (|*t*|, *t*‐test statistic; 95% CI, 95% confidence interval; DFd, degrees of freedom denominator; DFn, degrees of freedom numerator; *F*, F‐distribution; SE, standard error). Extended intercept tables for the complete DM1 cohort model are provided in Table S3 and for the CDM cohort in Table S4.

Given the numerous significant correlations between [MBNL]_inferred_ and many of the clinical outcomes assessed above, we sought to determine if clinical outcome measures could accurately predict [MBNL]_inferred_ via multiple linear regression modeling. Measures significantly correlated with [MBNL]_inferred_ in the complete DM1 cohort inclusive of both adults and children ( Figs. 2, 3, 4) were utilized in the “All – DM1 and CDM” model. Multiple linear regression analysis using all available sampled outcome measures led to a model that included 45 participants and had an adjusted *r* ^2^ = 0.6723 whereby the developed model was able to account for the majority of the variance in [MBNL]_inferred_ (Fig. 5A,C).

Next, we sought to build an equation to accurately predict [MBNL]_inferred_ in only children with CDM utilizing significantly correlated clinical outcome measures when this participant group was evaluated independently (Figs. 2, 3, 4). Multiple linear regression analysis using all available sampled CDM outcome measures led to a model that included 13 CDM participants and had an adjusted *r* ^2^ = 0.5875 (Fig. 5B,D). Despite the reduced sample size, the model still managed to moderately predict [MBNL]_inferred_ in children with CDM. Overall, these analyses indicate that clinical outcome measures of myotonia, motor function, and strength correlate significantly with MBNL‐dependent mis‐splicing within all participants with DM1 independent of age of disease onset. Additionally, these measures can be used in patients with DM1 to accurately predict disease‐associated patterns of mis‐splicing in skeletal muscle.

### 12‐month physical function can be predicted using baseline physical function measures and [MBNL]_inferred_

Given the strength of the correlations between [MBNL]_inferred_ and several of the clinical outcomes assessed above, we aimed to determine if future clinical performance could be accurately predicted using baseline [MBNL]_inferred_ values and baseline clinical performance assessments in adults with DM1 and in children with CDM. We first looked at the outcome measures that had the strongest correlations with [MBNL]_inferred_ values within the complete cross‐sectional cohort and in children with CDM independently – 10‐meter walk/running speed and ADF strength.

**Figure 6:**
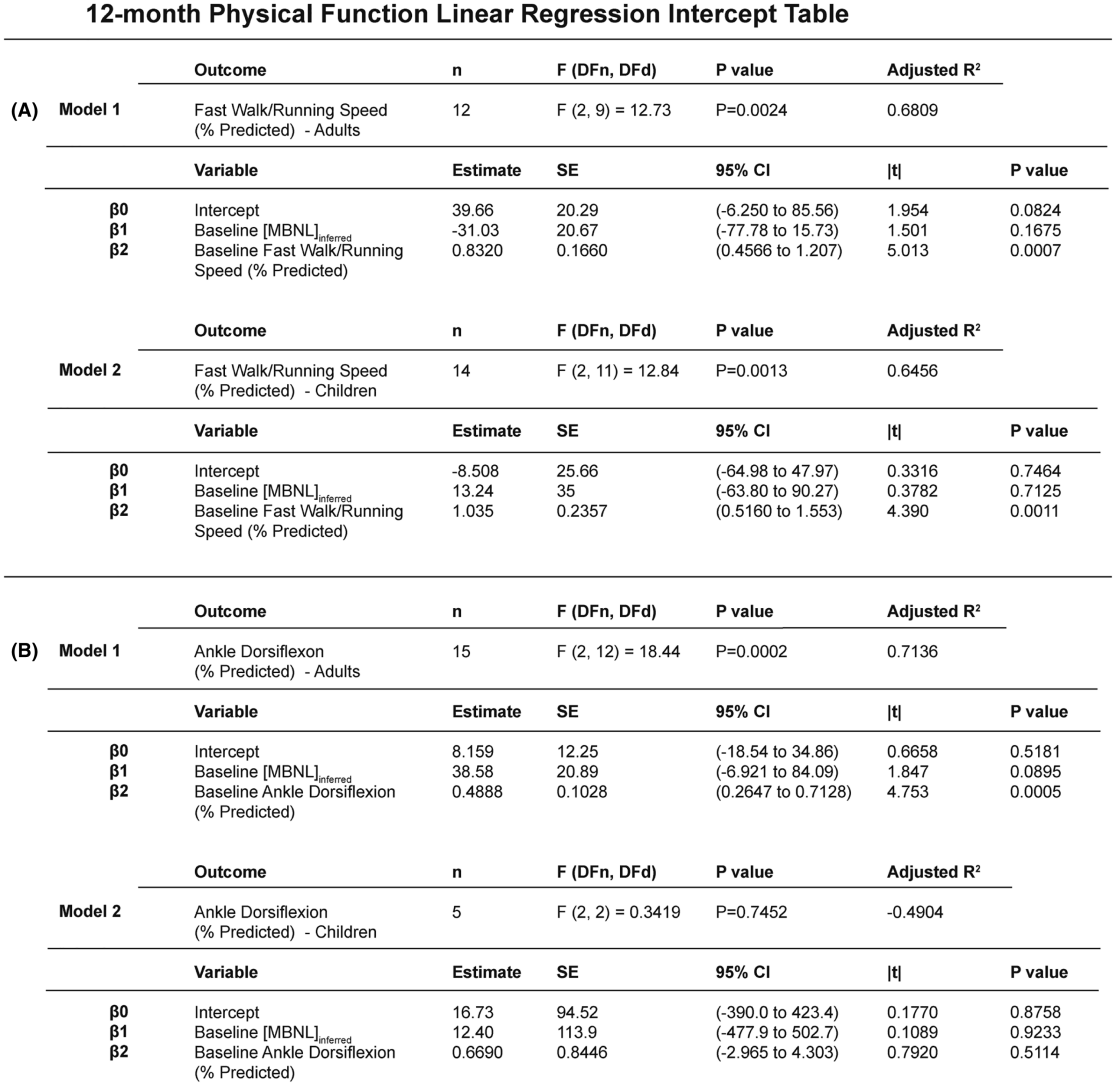
Multiple linear regression predictive models of 12‐month ankle dorsiflexion strength and 10‐meter walk/running speed in DM1 participants. Intercept table of multiple linear regression models for (A) 12‐month walk/running speed (% predicted) and (B) 12‐month ankle dorsiflexion strength (% predicted) using baseline [MBNL]_inferred_ values and baseline physical function values. Multiple statistical elements are reported (|*t*|, *t*‐test statistic; 95% CI, 95% confidence interval; DFd, degrees of freedom denominator; DFn, degrees of freedom numerator; *F*, *F*‐distribution; SE, standard error). Extended intercept tables for the 12‐month walk/running speed models and 12‐month ankle dorsiflexion strength are in Tables S5 and S6, respectively.

Multiple linear regression analysis for prediction of 12‐month ambulation speed using baseline walk/running speed and [MBNL]_inferred_ in our cohort of adults with DM1 had an adjusted *r* ^2^ = 0.681 (Fig. 6A, Model 1). Modeling in children with CDM alone suggested that these baseline values were similarly predictive of 12‐month performance (adjusted *r* ^2^ = 0.646) (Fig. 6A, Model 2). Interestingly, while baseline [MBNL]_inferred_ did not contribute significantly to the predictive power of either model, it was trending toward significance in the model for adults, but not in the model for CDM (*p* = 0.1675 & *p* = 0.7125, respectively).

Multiple linear regression analysis for predicting 12‐month ADF strength using baseline values and [MBNL]_inferred_ in our cohort of adults with DM1 had an adjusted *r* ^2^ = 0.7126 (Fig. 6B, Model 1). The predictive power of the same model for just children with CDM was reduced (adjusted *r* ^2^ = −0.4904) (Fig. 6B, Model 2). Again, when we evaluated the contribution of baseline [MBNL]_inferred_ to our 12‐month ADF predictive model in children with CDM, it was much less significant (*p* = 0.9233) as compared to that for adults with DM1 (*p* = 0.0895).

**Figure 7:**
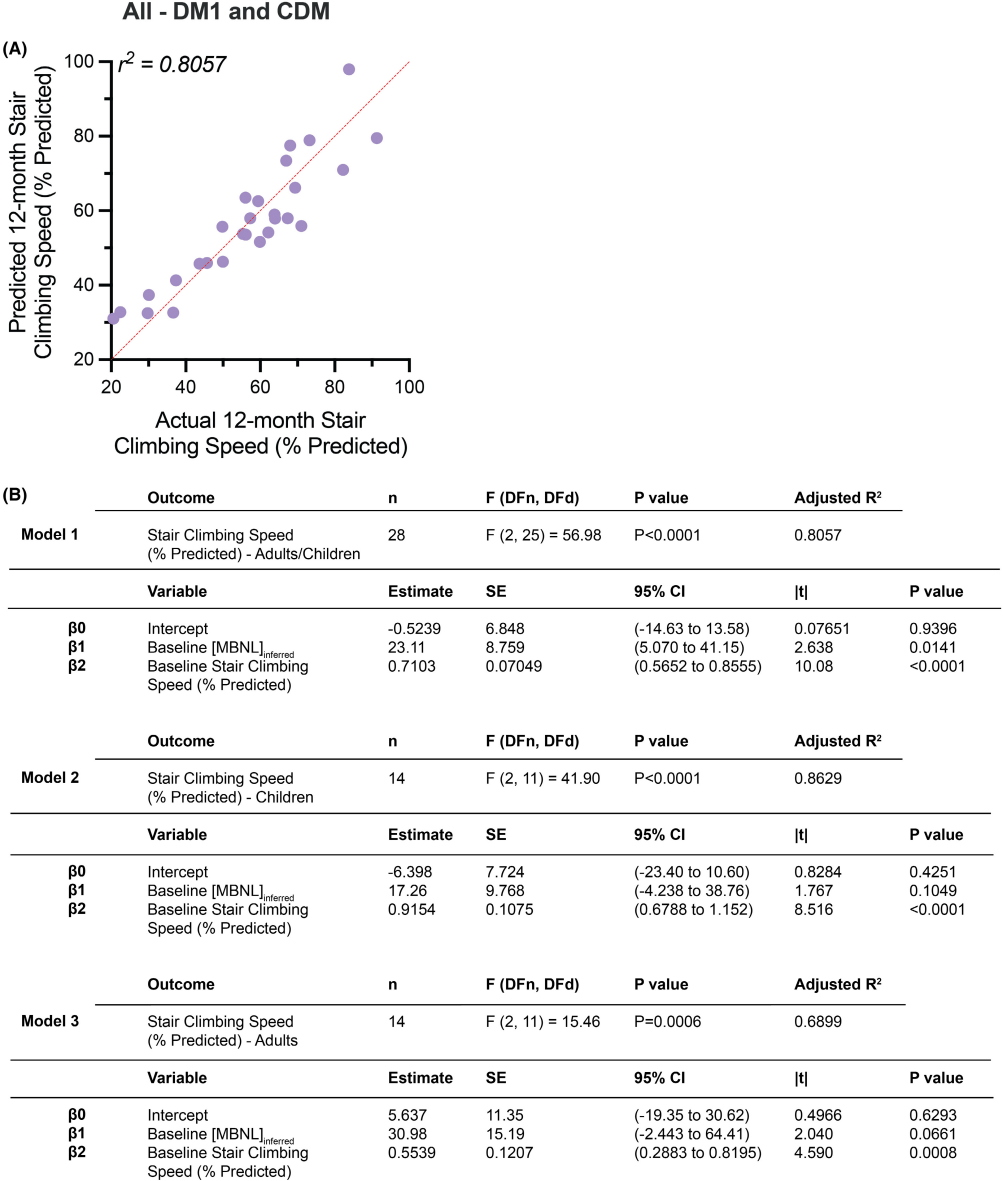
Multiple linear regression predictive modeling of 12‐month stair climbing speed in DM1 participants. (A) Multiple linear regression plot showing observed 12‐month stair climbing speed (% predicted) versus predicted 12‐month stair climbing speed (% predicted) for all DM1 individuals (adults and children). (B) Intercept table of multiple linear regression models for complete DM1 cohort (Model 1), CDM cohort alone (Model 2), and adult cohort alone (Model 3). Multiple statistical elements are reported (|*t*|, *t*‐test statistic; 95% CI, 95% confidence interval; DFd, degrees of freedom denominator; DFn, degrees of freedom numerator; *F*, *F*‐distribution; SE, standard error). Extended intercept tables for all three models are provided in Table S7.

Overall, of the seven motor function and strength outcome measures assessed, [MBNL]_inferred_ only contributed significantly to the predictive power of our 12‐month multiple linear regression models for stair climbing speed (Fig. 7). In our complete cross‐sectional cohort of both adults and children, baseline performance on this outcome assessment in combination with [MBNL]_inferred_ was able to accurately predict 12‐month performance (adjusted *r* ^2^ = 0.8057) (Fig. 7A,B, Model 1). Multiple linear regression analysis for predicting 12‐month stair climbing speed using baseline performance and [MBNL]_inferred_ in our cohort of CDM children alone had an adjusted *r* ^2^ = 0.8629 (Fig. 7B, Model 2), whereas the model for our cohort of adults with DM1 had an adjusted *r* ^2^ = 0.6899 (Fig. 7B, Model 3). The predictive utility of [MBNL]_inferred_ followed the same pattern observed above, whereby this measure of mis‐splicing contributed more to our adult model (*p* = 0.0661) as compared to the model for children with CDM (*p* = 0.1049).

## Discussion

In this study, we have identified associations between physical function and RNA mis‐splicing in skeletal muscle as captured by [MBNL]_inferred_, a composite metric of global alternative splicing dysregulation, in adults and children with DM1. This study describes significant correlations between mis‐splicing and timepoint‐matched assessments of clinical performance in children with CDM. Despite the availability of a small cohort of children and the wide age range assessed, the correlations observed across outcome assessments are surprisingly strong, underscoring theshared associations between alternative splicing dysregulation and phenotypic metrics in both adults with DM1 and children with CDM (Figs. 2, 3, 4).

Importantly, the regression model equations developed indicate that there are pathways forward in being able to predict levels of disease‐associated spliceopathy in skeletal muscle within individuals with DM1 using clinical outcome measures alone (Fig. 5). Given the variable degree of mis‐splicing in CDM, the ability to detect those children optimally suited for clinical trials by clinical outcome assessments alone may lessen the need for muscle biopsies in the future. The 12‐month predictive multiple linear regression modeling analyses using baseline measures of alternative splicing dysregulation and baseline functional outcome performance points to the prognostic utility of measures such as [MBNL]_inferred_ (Figs. 6 and 7). [MBNL]_inferred_ consistently trended toward significance in the 12‐month predictive models for adults with DM1; the small sample sizes (*n* ≤ 15) likely contributed to the lack of statistical significance observed. Contrary to adults, it appears that [MBNL]_inferred_ is unlikely to contribute to 12‐month predictive models in children with CDM due to the dynamic and variable pattern of splicing dysregulation occurring throughout development (Fig. 1). This is critical to keep in mind as we move forward in designing clinical trials for children with CDM, as RNA mis‐splicing in this patient population is dynamic and might confound results depending on age at assessment and the natural trajectory of CDM disease progression (CDM_infant_ vs. CDM_child_ vs. CDM_adolescent_).

Overall, there was a positive correlation between [MBNL]_inferred_ and motor/strength performance in all individuals with DM1 regardless of age of onset. Individuals with lower [MBNL]_inferred_ levels reflective of extensive dysregulated RNA splicing were not able to walk as far, run as fast, and took longer to climb stairs. In children with CDM, those with lower [MBNL]_inferred_ levels also exhibited reduced dexterity on the 9‐hole peg test, although this pattern was not seen in adults with DM1. Given the severe phenotype in infancy, it is reasonable to expect that this severe motor impairment may allow for detection of change on the 9‐hole peg test compared to the less severely affected adults with DM1. Most strikingly, individuals with higher [MBNL]_inferred_ levels performed more similarly to unaffected individuals as measured by quantitative muscle testing (knee extension, ankle dorsiflexors, grip strength). Importantly, these associations were not substantially impacted by choice of muscle biopsied or QMT testing method; [MBNL]_inferred_ was derived from vastus lateralis biopsies in children with CDM whereas distal tibialis anterior biopsies were utilized for adult‐onset DM1 participants. Despite this difference in muscle groups used to measure RNA mis‐splicing, children with CDM alone had a similar magnitude, if not stronger, association between [MBNL]_inferred_ and measures of distal strength (hand grip and ankle dorsiflexion) when compared to our analyses that included all DM1 participants. Altogether, the results suggest that these associations are more indicative of global alterations in muscle function and strength rather than these patterns being reflective of one specific muscle group.

Perhaps the most surprising finding of these analyses was the observed lack of correlation between [MBNL]_inferred_ or *CLCN1*/*CACNA1S* mis‐splicing levels with quantitative measures of myotonia in children with CDM. Previous work has shown that increased inclusion of *CLCN1* exon 7a leads to reduced density of CLCN1 within muscle fibers and myotonic discharges. Correction of this mis‐splicing via targeted antisense oligonucleotides is sufficient to rescue myotonia in DM1 mouse models.^45^ Mis‐splicing of the CaV1.1 calcium channel encoded by *CACNA1S* has also been linked with exacerbated myotonia in these model systems.^46^ Consistent with these reports, increased inclusion of *CLCN1* exon7a or exclusion of *CACNA1S* exon29 was associated moderately with longer vHOT_thumb_ times in all DM1 participants; no such associations of myotonia with causative RNA mis‐splicing events have been previously reported in human DM1 studies. However, these associations did not hold true in children with CDM. All CDM participants, regardless of sub‐cohort classification, [MBNL]_inferred_, or *CLCN1*/*CACNA1S* PSI values, had low vHOT_thumb_ times (<3.2 s). In fact, CDM_adolescent_ individuals who had nearly 100% *CLCN1* exon inclusion, like the most severely affected adults with DM1, possessed minimal quantitative myotonia. Similar patterns were observed for *CACNA1S* mis‐splicing. Altogether, these data suggest that while patterns of DM1 spliceopathy previously linked to myotonia are preserved in CDM skeletal muscle, these pediatric patients are not experiencing clinical myotonia in the same manner as adults with DM1. Future work will be required to evaluate the molecular mechanisms behind this lack of observable myotonia in the CDM population. In total, these data suggest that myotonia measures appear to be a poor outcome assessment for children with CDM, especially as correction of myotonia and the associated RNA splicing patterns have been proposed as early markers of therapeutic efficacy in DM1 clinical trials.

Previous work by our group has shown that children with CDM display a triphasic pattern of disease progression that is mirrored by changes in RNA mis‐splicing.^13^, ^28^ While the CDM participants evaluated here were sampled cross‐sectionally, the results captured in this study begin to verify that the triphasic pattern of alternative splicing dysregulation in CDM matches the observed changes in physical function. However, we were unable to fully validate this proposed pattern given the limited sample size and data availability within select CDM sub‐cohorts, notably CDM infants (<2 years). Few clinical measures were able to be assessed for these individuals due to the developmental constraints of this age group. Future directions include improving our clinical outcome measures to include infant‐specific strength/motor testing. Another challenge in obtaining larger sample sizes lies in the difficulty in obtaining reliable outcomes from patients with physical, cognitive, and behavioral impairments. Due to these challenges, it is unclear whether clinical outcome measures reflect true maximum physical ability, or rather a child's ability to understand and follow instructions. This could be especially relevant in measures such as the 9‐hole peg test. Future studies with expanded cohort sizes will need to be performed to assess the impacts of cognitive impairment as a potential confounding variable in physical outcome measure performance and identification of representative biomarkers that correlate with measures of cognitive function.

The development of disease‐modifying therapies for adults with DM1 has rapidly accelerated in recent years with many in early‐phase clinical trials. Given the shared genetic basis of disease, there is significant interest in extending these trials into the CDM population. However, a major limitation of these efforts has been insufficient understanding of the natural history of disease progression in children with CDM and how the molecular pathogenesis, particularly alternative splicing dysregulation, correlates with clinical outcome measures. This analysis connects measures of splicing dysregulation to a range of clinical outcome measures in a cross‐sectional cohort including both adult DM1 and CDM participants. Furthermore, the analyses here provide the foundation for determination of high‐performing clinical outcome assessments (COAs) for use in CDM clinical trials in older pediatric patients (>6 years). This work, in combination with our characterization of CDM spliceopathy across pediatric development,^28^ is vital in identifying the effective timing of therapeutic administration to children with CDM for clinical trial success whereby therapeutic benefit outpaces the natural dynamic changes in RNA mis‐splicing, especially given the rapid improvement observed in the first years of life (Fig. 1). The regression models developed in this study may assist in the refinement of clinical trial design and offer a noninvasive methodology for prediction of spliceopathy using COAs alone when screening for trial inclusion. They may also reduce reliance on muscle biopsies to assess target engagement of therapeutic agents. The strength of these predictive equations can be improved through replication and validation in a secondary DM1 cohort. Additionally, these predicative equations are based on inferences of free [MBNL] in skeletal muscle and may not be reflective of disease severity and clinical performance for non‐musculoskeletal metrics. Overall, these results provide the framework for both the utility of a muscle‐based biomarker and clinical trial design in children with CDM.

## Author Contributions

Conception and design of study: JMH, MAH, and NEJ. Acquisition and analysis of data: JMH, MP, KB, KI, AB, ASJ, KB, JD, MJM, JNB, KMC, JB, MK, NEJ, and MAH. Drafting a significant portion of manuscript: JMH, MK, NEJ, and MAH. Please also see the supplementary file labeled “DMCRN Consortium Members.”

## Conflict of Interest

Julia M. Hartman, Marina Provenzano, Kameron Bates, Kobe Ikegami, Amanda Butler, Aileen S. Jones, Kiera N. Berggren, Marnee J. McKay, Jennifer N. Baldwin, and Kayla M.D. Cornett – None. Jeanne Dekdebrun – Consultation for Avidity Biosciences, Dyne Therapeutics, Vertex, Lupin, Arthex, PepGen and Trins. Joshua Burns – Research Support from the University of Sydney, Sydney Children's Hospitals Network, Australian Government (NHMRC#2015970, MRFF#1152226), United States Government (NIH NINDS#1U01NS109403, NIH NCATS/NINDS# U54NS065712), Muscular Dystrophy Association, American Orthotic and Prosthetic Association, Charcot Marie Tooth Association and Charcot Marie Tooth Australia. Scientific Advisory Board fees from Faculty of Medicine Siriraj Hospital Mahidol University Thailand; Department of Rehabilitation Sciences, The Hong Kong Polytechnic University; Hereditary Neuropathy Foundation. Consulted for DTx Pharma, Applied Therapeutics, Pharnext. Michael Kiefer – Has provided consultation for Aspa therapeutics. Nicholas E. Johnson – He has received grant funding from NINDS (R01NS104010, U01NS124974), NCATS (R21TR003184), CDC (U01DD001242) and the FDA (7R01FD006071). He receives royalties from the CCMDHI and the CMTHI. He receives research funds from Novartis, Takeda, PepGen, Sanofi Genzyme, Dyne, Vertex Pharmaceuticals, Fulcrum Therapeutics, AskBio, ML Bio, and Sarepta. He has provided consultation for Arthex, Angle Therapeutics, Juvena, Rgenta, PepGen, AMO Pharma, Takeda, Design, Dyne, AskBio, Avidity, and Vertex Pharmaceuticals. Melissa A. Hale – She has provided consultation for Juvena and Arrakis Therapeutics.

## Supporting information


Data S1.

Appendix S1.

## Data Availability

RNA sequencing data used to define [MBNL]_inferred_ are available in Sequence Read Archive at https://www.ncbi.nlm.nih.gov/sra in the following BioProjects: [PRJNA1079722], [PRJNA830511], and [PRJNA1151618]. Associated BioProject and SRA accession numbers for each sample are listed in Table S1.
