# Supplementary material for: RNA mis‐splicing in children with congenital myotonic dystrophy is associated with physical function
Source: Ann Clin Transl Neurol. 2024 Oct 25;11(12):3175–91. doi: 10.1002/acn3.52224 (PMC11651218; doi:10.1002/acn3.52224)
Supplement: Supplementary file 2 — Appendix S1. [file ACN3-11-3175-s001.zip › Supplemental Table 1 - MBNL Demographics _ Updated.docx]

Supplemental Table 1

| **Sample ID** | **Biopsy Location** | **Cohort** | **CDM Sub-cohort** | **Visit** | **Sex** | **Age at Biopsy (yrs.)** | **CTG Repeat** | **[MBNL]_inferred_** | **Bioproject ID** | **SRA**  **Accession**  **Number** |
| --- | --- | --- | --- | --- | --- | --- | --- | --- | --- | --- |
| CDM-01* | vastus lateralis | CDM | CDM infant | 0 | M | 0.04 | 1500 | 0.015 | PRJNA830511 | SRR18858698 |
| CDM-02 | vastus lateralis | CDM | CDM infant | 0 | M | 0.125 |  | 0.013 | PRJNA830511 | SRR18858697 |
| CDM-03 | vastus lateralis | CDM | CDM infant | 0 | M | 0.25 |  | 0.021 | PRJNA830511 | SRR18858680 |
| CDM-04 | vastus lateralis | CDM | CDM infant | 0 | F | 1 | 1700 | 0.139 | PRJNA830511 | SRR18858679 |
| CDM-05 | vastus lateralis | CDM | CDM infant | 0 | M | 1 |  | 0.681 | PRJNA1151618 | SRR30351191 |
| CDM-06 | vastus lateralis | CDM | CDM infant | 0 | M | 2 |  | 0.83 | PRJNA1151618 | SRR30351190 |
| CDM-07 | vastus lateralis | CDM | CDM infant | 0 | M | 0.92 |  | 0.349 | PRJNA1151618 | SRR30351188 |
| CDM-08 | vastus lateralis | CDM | CDM child | 0 | M | 7 |  | 0.441 | PRJNA1151618 | SRR30351193 |
| CDM-09 | vastus lateralis | CDM | CDM child | 0 | M | 3 |  | 0.907 | PRJNA1151618 | SRR30351189 |
| CDM-10 | vastus lateralis | CDM | CDM child | 0 | F | 2.5 | 1500 | 0.522 | PRJNA830511 | SRR18858678 |
| CDM-11 | vastus lateralis | CDM | CDM child | 0 | M | 2.5 | 750 | 0.778 | PRJNA830511 | SRR18858677 |
| CDM-12 | vastus lateralis | CDM | CDM child | 0 | M | 5 | 1000 | 0.493 | PRJNA830511 | SRR18858676 |
| CDM-13 | vastus lateralis | CDM | CDM child | 0 | M | 5 | 1060 | 0.519 | PRJNA830511 | SRR18858675 |
| CDM-14 | vastus lateralis | CDM | CDM child | 0 | M | 5 | 938 | 0.643 | PRJNA830511 | SRR18858674 |
| CDM-15 | vastus lateralis | CDM | CDM child | 0 | M | 5 | 1000 | 0.674 | PRJNA830511 | SRR18858673 |
| CDM-16 | vastus lateralis | CDM | CDM child | 0 | F | 5 | 550 | 0.92 | PRJNA830511 | SRR18858672 |
| CDM-17 | vastus lateralis | CDM | CDM child | 0 | F | 6 | 1450 | 0.757 | PRJNA830511 | SRR18858671 |
| CDM-18 | vastus lateralis | CDM | CDM child | 0 | M | 6 | 1300 | 0.536 | PRJNA830511 | SRR18858670 |
| CDM-19 | vastus lateralis | CDM | CDM child | 0 | M | 6 | 465 | 0.916 | PRJNA830511 | SRR18858669 |
| CDM-20 | vastus lateralis | CDM | CDM child | 0 | M | 6 | 1505 | 0.715 | PRJNA830511 | SRR18858668 |
| CDM-21 | vastus lateralis | CDM | CDM child | 0 | M | 7 | 950 | 0.826 | PRJNA830511 | SRR18858667 |
| CDM-22 | vastus lateralis | CDM | CDM adolescent | 0 | M | 8 | 986 | 0.702 | PRJNA830511 | SRR18858666 |
| CDM-23* | vastus lateralis | CDM | CDM adolescent | 0 | M | 8 | 1500 | 0.577 | PRJNA830511 | SRR18858665 |
| CDM-24 | vastus lateralis | CDM | CDM adolescent | 0 | F | 8 | 773 | 0.903 | PRJNA830511 | SRR18858648 |
| CDM-25 | vastus lateralis | CDM | CDM adolescent | 0 | M | 8 | 1500 | 0.214 | PRJNA830511 | SRR18858647 |
| CDM-26 | vastus lateralis | CDM | CDM adolescent | 0 | F | 8 | 1473 | 0.264 | PRJNA830511 | SRR18858646 |
| CDM-27 | vastus lateralis | CDM | CDM adolescent | 0 | F | 8 | 1800 | 0.729 | PRJNA830511 | SRR18858645 |
| CDM-28 | vastus lateralis | CDM | CDM child | 0 | F | 7 | 983 | 0.712 | PRJNA830511 | SRR18858644 |
| CDM-29 | vastus lateralis | CDM | CDM adolescent | 0 | F | 8 |  | 0.608 | PRJNA830511 | SRR18858643 |
| CDM-30** | vastus lateralis | CDM | CDM adolescent | 0 | M | 9 | 854 | 0.933 | PRJNA830511 | SRR18858642 |
| CDM-31 | vastus lateralis | CDM | CDM adolescent | 0 | F | 10 |  | 0.306 | PRJNA830511 | SRR18858641 |
| CDM-32 | vastus lateralis | CDM | CDM adolescent | 0 | M | 10 | 1200 | 0.824 | PRJNA830511 | SRR18858640 |
| CDM-33 | vastus lateralis | CDM | CDM adolescent | 0 | M | 11 | 1050 | 0.787 | PRJNA830511 | SRR18858639 |
| CDM-34 | vastus lateralis | CDM | CDM adolescent | 0 | F | 11 | 1136 | 0.544 | PRJNA830511 | SRR18858638 |
| CDM-35 | soleus | CDM | CDM adolescent | 0 | F | 11 |  | 0.151 | PRJNA830511 | SRR18858637 |
| CDM-36 | vastus lateralis | CDM | CDM adolescent | 0 | F | 12 | 2530 | 0.275 | PRJNA830511 | SRR18858636 |
| CDM-37*** | vastus lateralis | CDM | CDM adolescent | 0 | F | 12 | 710 | 0.727 | PRJNA830511 | SRR18858635 |
| CDM-38 | vastus lateralis | CDM | CDM adolescent | 0 | F | 14 | 1480 | 0.018 | PRJNA830511 | SRR18858634 |
| CDM-39 | vastus lateralis | CDM | CDM adolescent | 0 | M | 14 | 450 | 0.863 | PRJNA830511 | SRR18858633 |
| CDM-40 | vastus lateralis | CDM | CDM adolescent | 0 | F | 16 |  | 0.352 | PRJNA830511 | SRR18858628 |
| CDM-41*** | vastus lateralis | CDM | CDM adolescent | 0 | F | 16 | 710 | 0.748 | PRJNA830511 | SRR18858627 |
| CDM-42 | vastus lateralis | CDM | CDM adolescent | 0 | M | 16 |  | 0.903 | PRJNA1151618 | SRR30351195 |
| CDM-43** | vastus lateralis | CDM | CDM adolescent | 0 | M | 13 |  | 0.8 | PRJNA1151618 | SRR30351194 |
| CDM-44 |  | CDM | CDM infant | 2 | M | 1 | 1500 |  |  |  |
| CDM-45 |  | CDM | CDM infant | 2 | M | 1 |  |  |  |  |
| CDM-46 |  | CDM | CDM infant | 2 | M | 1 |  |  |  |  |
| CDM-47 |  | CDM | CDM infant | 2 | F | 2 | 1700 |  |  |  |
| CDM-48 |  | CDM | CDM child | 2 | F | 3 | 1500 |  |  |  |
| CDM-49 |  | CDM | CDM child | 2 | M | 4 |  |  |  |  |
| CDM-50 |  | CDM | CDM child | 2 | M | 6 | 1000 |  |  |  |
| CDM-51 |  | CDM | CDM child | 2 | M | 6 | 1060 |  |  |  |
| CDM-52 |  | CDM | CDM child | 2 | M | 6 | 938 |  |  |  |
| CDM-53 |  | CDM | CDM child | 2 | M | 6 | 1000 |  |  |  |
| CDM-54 |  | CDM | CDM child | 2 | F | 6 | 550 |  |  |  |
| CDM-55 |  | CDM | CDM child | 2 | F | 7 | 1450 |  |  |  |
| CDM-56 |  | CDM | CDM child | 2 | M | 7 | 1300 |  |  |  |
| CDM-57 |  | CDM | CDM child | 2 | M | 7 | 465 |  |  |  |
| CDM-58 |  | CDM | CDM child | 2 | M | 7 | 1505 |  |  |  |
| CDM-59 |  | CDM | CDM child | 2 | M | 8 | 950 |  |  |  |
| CDM-60 |  | CDM | CDM child | 2 | M | 9 | 986 |  |  |  |
| CDM-61 |  | CDM | CDM adolescent | 2 | M | 9 | 1500 |  |  |  |
| CDM-62 |  | CDM | CDM adolescent | 2 | F | 9 | 773 |  |  |  |
| CDM-63 |  | CDM | CDM adolescent | 2 | M | 9 | 1500 |  |  |  |
| CDM-64 |  | CDM | CDM adolescent | 2 | F | 9 | 1473 |  |  |  |
| CDM-65 |  | CDM | CDM adolescent | 2 | F | 9 | 1800 |  |  |  |
| CDM-66 |  | CDM | CDM adolescent | 2 | F | 8 | 983 |  |  |  |
| CDM-67 |  | CDM | CDM adolescent | 2 | F | 9 |  |  |  |  |
| CDM-68 |  | CDM | CDM adolescent | 2 | M | 10 | 854 |  |  |  |
| CDM-69 |  | CDM | CDM adolescent | 2 | F | 11 |  |  |  |  |
| CDM-70 |  | CDM | CDM adolescent | 2 | M | 11 | 1200 |  |  |  |
| CDM-71 |  | CDM | CDM adolescent | 2 | M | 12 | 1050 |  |  |  |
| CDM-72 |  | CDM | CDM adolescent | 2 | F | 12 | 1136 |  |  |  |
| CDM-73 |  | CDM | CDM adolescent | 2 | F | 12 |  |  |  |  |
| CDM-74 |  | CDM | CDM adolescent | 2 | F | 13 | 2530 |  |  |  |
| CDM-75 |  | CDM | CDM adolescent | 2 | F | 13 | 710 |  |  |  |
| CDM-76 |  | CDM | CDM adolescent | 2 | F | 15 | 1480 |  |  |  |
| CDM-77 |  | CDM | CDM adolescent | 2 | M | 15 | 450 |  |  |  |
| DM1-01 | tibialis anterior | DM1 |  | 0 | F | 34 | 340 | 0.58 | PRJNA1079722 | SRR28067330 |
| DM1-02 | tibialis anterior | DM1 |  | 0 | F | 31 | 350 | 0.563 | PRJNA1079722 | SRR28067318 |
| DM1-03 | tibialis anterior | DM1 |  | 0 | M | 43 |  | 0.231 | PRJNA1079722 | SRR28067275 |
| DM1-04 | tibialis anterior | DM1 |  | 0 | M | 37 | 350 | 0.507 | PRJNA1079722 | SRR28067312 |
| DM1-05 | tibialis anterior | DM1 |  | 0 | F | 33 |  | 0.386 | PRJNA1079722 | SRR28067290 |
| DM1-06 | tibialis anterior | DM1 |  | 0 | M | 28 |  | 0.634 | PRJNA1079722 | SRR28067248 |
| DM1-07 | tibialis anterior | DM1 |  | 0 | F | 30 |  | 0.446 | PRJNA1079722 | SRR28067257 |
| DM1-08 | tibialis anterior | DM1 |  | 0 | F | 43 |  | 0.036 | PRJNA1079722 | SRR28067327 |
| DM1-09 | tibialis anterior | DM1 |  | 0 | M | 28 | 505 | 0.221 | PRJNA1079722 | SRR28067325 |
| DM1-10 | tibialis anterior | DM1 |  | 0 | M | 43 | 893 | 0.494 | PRJNA1079722 | SRR28067323 |
| DM1-11 | tibialis anterior | DM1 |  | 0 | F | 39 | 600 | 0.303 | PRJNA1079722 | SRR28067322 |
| DM1-12 | tibialis anterior | DM1 |  | 0 | M | 41 | 866 | 0.364 | PRJNA1079722 | SRR28067320 |
| DM1-13 | tibialis anterior | DM1 |  | 0 | M | 41 | 746 | 0.38 | PRJNA1079722 | SRR28067317 |
| DM1-14 | tibialis anterior | DM1 |  | 0 | F | 54 |  | 0.133 | PRJNA1079722 | SRR28067316 |
| DM1-15 | tibialis anterior | DM1 |  | 0 | M | 49 |  | 0.33 | PRJNA1079722 | SRR28067282 |
| DM1-16 | tibialis anterior | DM1 |  | 0 | F | 56 |  | 0.456 | PRJNA1079722 | SRR28067280 |
| DM1-17 | tibialis anterior | DM1 |  | 0 | F | 57 | 179 | 0.081 | PRJNA1079722 | SRR28067279 |
| DM1-18 | tibialis anterior | DM1 |  | 0 | M | 36 | 677 | 0.208 | PRJNA1079722 | SRR28067277 |
| DM1-19 | tibialis anterior | DM1 |  | 0 | M | 38 | 720 | 0.032 | PRJNA1079722 | SRR28067274 |
| DM1-20 | tibialis anterior | DM1 |  | 0 | M | 35 |  | 0.025 | PRJNA1079722 | SRR28067272 |
| DM1-21 | tibialis anterior | DM1 |  | 0 | M | 41 | 441 | 0.054 | PRJNA1079722 | SRR28067270 |
| DM1-22 | tibialis anterior | DM1 |  | 1 | F | 35 | 340 | 0.595 | PRJNA1079722 | SRR28067329 |
| DM1-23 | tibialis anterior | DM1 |  | 1 | M | 44 | 350 | 0.437 | PRJNA1079722 | SRR28067301 |
| DM1-24 | tibialis anterior | DM1 |  | 1 | M | 29 |  | 0.478 | PRJNA1079722 | SRR28067237 |
| DM1-25 | tibialis anterior | DM1 |  | 1 | F | 30 |  | 0.416 | PRJNA1079722 | SRR28067328 |
| DM1-26 | tibialis anterior | DM1 |  | 1 | F | 44 |  | 0.071 | PRJNA1079722 | SRR28067326 |
| DM1-27 | tibialis anterior | DM1 |  | 1 | M | 29 | 505 | 0.197 | PRJNA1079722 | SRR28067324 |
| DM1-28 | tibialis anterior | DM1 |  | 1 | F | 39 | 600 | 0.015 | PRJNA1079722 | SRR28067321 |
| DM1-29 | tibialis anterior | DM1 |  | 1 | M | 41 | 866 | 0.219 | PRJNA1079722 | SRR28067319 |
| DM1-30 | tibialis anterior | DM1 |  | 1 | F | 55 |  | 0.291 | PRJNA1079722 | SRR28067315 |
| DM1-31 | tibialis anterior | DM1 |  | 1 | M | 49 |  | 0.038 | PRJNA1079722 | SRR28067281 |
| DM1-32 | tibialis anterior | DM1 |  | 1 | F | 57 | 179 | 0.128 | PRJNA1079722 | SRR28067278 |
| DM1-33 | tibialis anterior | DM1 |  | 1 | M | 37 | 677 | 0.092 | PRJNA1079722 | SRR28067276 |
| DM1-34 | tibialis anterior | DM1 |  | 1 | M | 38 | 720 | 0.052 | PRJNA1079722 | SRR28067273 |
| DM1-35 | tibialis anterior | DM1 |  | 1 | M | 36 |  | 0.042 | PRJNA1079722 | SRR28067271 |
| DM1-36 | tibialis anterior | DM1 |  | 1 | M | 42 | 441 | 0.034 | PRJNA1079722 | SRR28067269 |
| DM1-37 | tibialis anterior | DM1 |  | 0 | F | 31 |  | 0.83 | PRJNA1079722 | SRR29688962 |
| DM1-38 | tibialis anterior | DM1 |  | 0 | M | 35 |  | 0.825 | PRJNA1079722 | SRR29688961 |
| DM1-39 | tibialis anterior | DM1 |  | 0 | F | 42 |  | 0.537 | PRJNA1079722 | SRR28067268 |
| DM1-40 | tibialis anterior | DM1 |  | 0 | F | 42 | 313 | 0.488 | PRJNA1079722 | SRR28067314 |
| DM1-41 | tibialis anterior | DM1 |  | 0 | F | 41 | 133 | 0.638 | PRJNA1079722 | SRR28067311 |
| DM1-42 | tibialis anterior | DM1 |  | 0 | F | 45 |  | 0.672 | PRJNA1079722 | SRR28067309 |
| DM1-43 | tibialis anterior | DM1 |  | 0 | F | 33 | 170 | 0.511 | PRJNA1079722 | SRR28067307 |
| DM1-44 | tibialis anterior | DM1 |  | 0 | F | 38 |  | 0.503 | PRJNA1079722 | SRR28067306 |
| DM1-45 | tibialis anterior | DM1 |  | 0 | F | 38 | 300 | 0.627 | PRJNA1079722 | SRR28067304 |
| DM1-46 | tibialis anterior | DM1 |  | 0 | M | 45 | 108 | 0.956 | PRJNA1079722 | SRR28067302 |
| DM1-47 | tibialis anterior | DM1 |  | 0 | F | 21 |  | 0.749 | PRJNA1079722 | SRR28067299 |
| DM1-48 | tibialis anterior | DM1 |  | 0 | M | 20 |  | 0.517 | PRJNA1079722 | SRR28067297 |
| DM1-49 | tibialis anterior | DM1 |  | 0 | F | 42 |  | 0.56 | PRJNA1079722 | SRR28067295 |
| DM1-50 | tibialis anterior | DM1 |  | 0 | F | 62 | 336 | 0.479 | PRJNA1079722 | SRR28067294 |
| DM1-51 | tibialis anterior | DM1 |  | 0 | F | 44 |  | 0.392 | PRJNA1079722 | SRR28067292 |
| DM1-52 | tibialis anterior | DM1 |  | 0 | F | 32 | 750 | 0.425 | PRJNA1079722 | SRR28067291 |
| DM1-53 | tibialis anterior | DM1 |  | 0 | M | 31 |  | 0.748 | PRJNA1079722 | SRR28067288 |
| DM1-54 | tibialis anterior | DM1 |  | 0 | F | 21 | 300 | 0.729 | PRJNA1079722 | SRR28067286 |
| DM1-55 | tibialis anterior | DM1 |  | 0 | F | 44 |  | 0.874 | PRJNA1079722 | SRR28067284 |
| DM1-56 | tibialis anterior | DM1 |  | 0 | F | 38 | 200 | 0.491 | PRJNA1079722 | SRR28067251 |
| DM1-57 | tibialis anterior | DM1 |  | 0 | M | 69 | 98 | 0.772 | PRJNA1079722 | SRR28346193 |
| DM1-58 | tibialis anterior | DM1 |  | 1 | F | 42 |  | 0.604 | PRJNA1079722 | SRR28067267 |
| DM1-59 | tibialis anterior | DM1 |  | 1 | F | 42 | 313 | 0.098 | PRJNA1079722 | SRR28067313 |
| DM1-60 | tibialis anterior | DM1 |  | 1 | F | 41 | 133 | 0.651 | PRJNA1079722 | SRR28067310 |
| DM1-61 | tibialis anterior | DM1 |  | 1 | F | 45 |  | 0.928 | PRJNA1079722 | SRR28067308 |
| DM1-62 | tibialis anterior | DM1 |  | 1 | F | 38 |  | 0.289 | PRJNA1079722 | SRR28067305 |
| DM1-63 | tibialis anterior | DM1 |  | 1 | F | 38 | 300 | 0.358 | PRJNA1079722 | SRR28067303 |
| DM1-64 | tibialis anterior | DM1 |  | 1 | M | 45 | 108 | 0.797 | PRJNA1079722 | SRR28067300 |
| DM1-65 | tibialis anterior | DM1 |  | 1 | F | 21 |  | 0.563 | PRJNA1079722 | SRR28067298 |
| DM1-66 | tibialis anterior | DM1 |  | 1 | M | 20 |  | 0.368 | PRJNA1079722 | SRR28067296 |
| DM1-67 | tibialis anterior | DM1 |  | 1 | F | 62 | 336 | 0.412 | PRJNA1079722 | SRR28067293 |
| DM1-68 | tibialis anterior | DM1 |  | 1 | F | 32 | 750 | 0.433 | PRJNA1079722 | SRR28067289 |
| DM1-69 | tibialis anterior | DM1 |  | 1 | M | 31 |  | 0.784 | PRJNA1079722 | SRR28067287 |
| DM1-70 | tibialis anterior | DM1 |  | 1 | F | 21 | 300 | 0.707 | PRJNA1079722 | SRR28067285 |
| DM1-71 | tibialis anterior | DM1 |  | 1 | F | 44 |  | 0.819 | PRJNA1079722 | SRR28067283 |
| DM1-72 | tibialis anterior | DM1 |  | 1 | F | 38 | 200 | 0.412 | PRJNA1079722 | SRR28067250 |
| DM1-73 | tibialis anterior | DM1 |  | 1 | M | 69 | 98 | 0.722 | PRJNA1079722 | SRR28067247 |
| DM1-74 |  | DM1 |  | 2 | F | 43 |  |  |  |  |
| DM1-75 |  | DM1 |  | 2 | F | 43 | 313 |  |  |  |
| DM1-76 |  | DM1 |  | 2 | F | 42 | 133 |  |  |  |
| DM1-77 |  | DM1 |  | 2 | F | 34 |  |  |  |  |
| DM1-78 |  | DM1 |  | 2 | F | 39 | 108 |  |  |  |
| DM1-79 |  | DM1 |  | 2 | M | 46 |  |  |  |  |
| DM1-80 |  | DM1 |  | 2 | F | 22 |  |  |  |  |
| DM1-81 |  | DM1 |  | 2 | M | 21 | 336 |  |  |  |
| DM1-82 |  | DM1 |  | 2 | F | 63 |  |  |  |  |
| DM1-83 |  | DM1 |  | 2 | F | 45 | 300 |  |  |  |
| DM1-84 |  | DM1 |  | 2 | F | 33 |  |  |  |  |
| DM1-85 |  | DM1 |  | 2 | M | 32 | 200 |  |  |  |
| DM1-86 |  | DM1 |  | 2 | F | 22 | 98 |  |  |  |
| DM1-87 |  | DM1 |  | 2 | F | 39 |  |  |  |  |
| DM1-88 |  | DM1 |  | 2 | M | 70 |  |  |  |  |
