# Supplementary figures and images for: RNA mis‐splicing in children with congenital myotonic dystrophy is associated with physical function
Source: Ann Clin Transl Neurol. 2024 Oct 25;11(12):3175–91. doi: 10.1002/acn3.52224 (PMC11651218; doi:10.1002/acn3.52224)

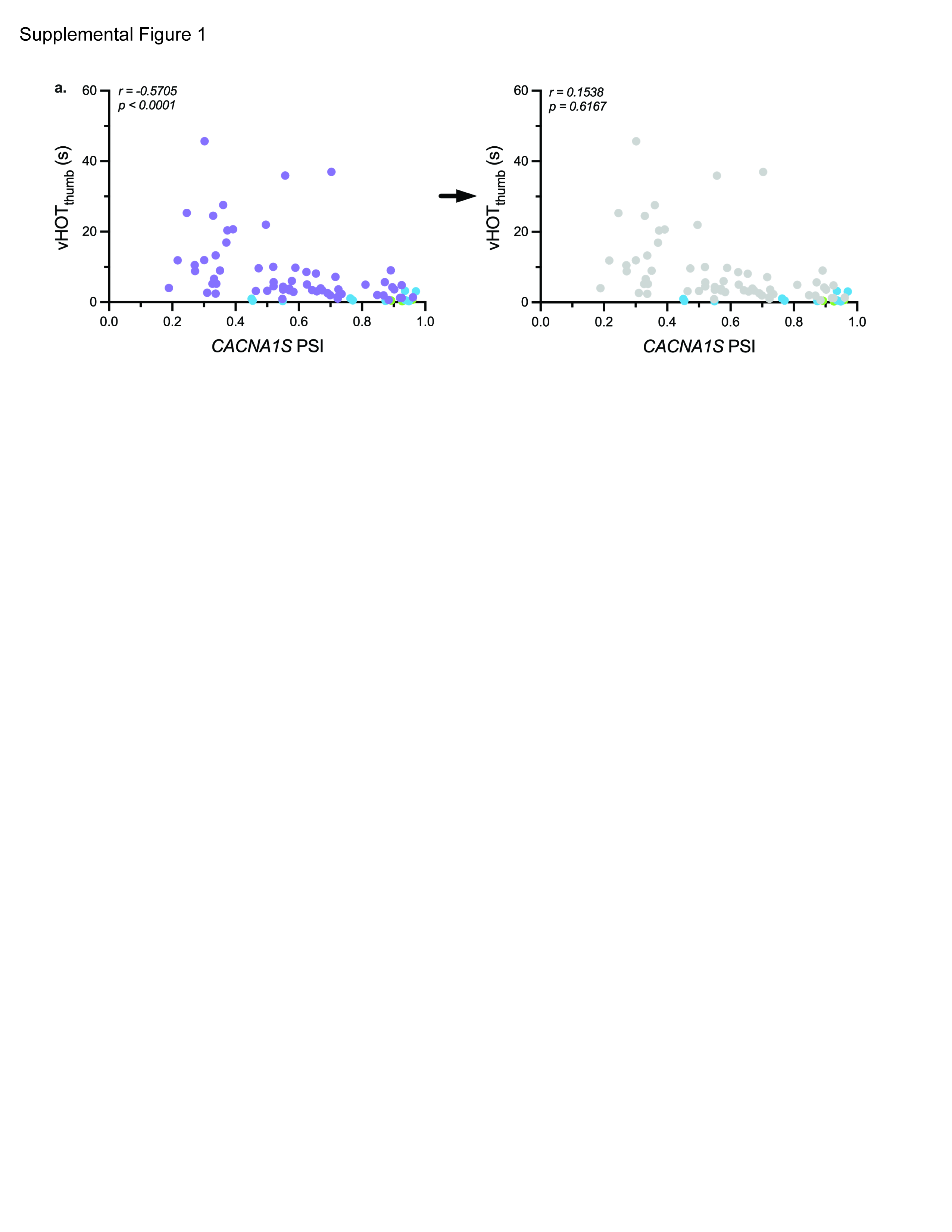

Supplement: Supplementary file 2 — Appendix S1. [file ACN3-11-3175-s001.zip › Sup Figure 1.tif]
